# Chronic insomnia: are patients also suffering from PTSD symptoms?

**DOI:** 10.3389/frsle.2023.1207232

**Published:** 2023-09-06

**Authors:** Emma Lardant, François Vialatte, Céline Ramdani, Frédéric Chauveau, Caroline Gauriau, Léna Storms, Marion Trousselard, Damien Léger

**Affiliations:** ^1^Institut de Recherche Biomédicale des Armées, Brétigny-sur-Orge, France; ^2^Institut Pi-Psy, Draveil, France; ^3^Assistance Publique – Hôpitaux de Paris (APHP), Hôtel-Dieu, Centre du sommeil et de la Vigilance, Paris, France; ^4^Université Paris Cité, VIFASOM (EA 7330 Vigilance, Fatigue, Sommeil et Santé Publique), Paris, France; ^5^Ecole du Val de Grâce, Paris, France; ^6^Adaptation, mesure et prévention en santé (APEMAC/EPSAM), Metz, France

**Keywords:** insomnia, post-traumatic stress disorder, trauma history, stress, sex

## Abstract

**Introduction:**

Insomnia is highly prevalent in the general population, and is commonly associated with somatic and psychiatric comorbidities. However, its origins remain poorly-understood. Recently, adverse childhood events (ACE), including traumatic experiences, have been found to be significantly associated with both insomnia and Post-Traumatic Stress Disorders (PTSD). Many patients with PTSD suffer from sleep disorders. However, we know much less about traumatic childhood experiences in patients with insomnia and PTSD.

**Methods:**

Our exploratory study investigated a cohort of 43 patients (14 males, 29 females) clinically diagnosed with chronic insomnia at a sleep center, and systematically evaluated their condition using the trauma history questionnaire (THQ), and the PTSD checklist (PCL-5).

**Results:**

Our results show that 83.72% of insomnia patients reported at least one traumatic event, while the prevalence of PTSD symptoms was 53.49%. For 11.6% of patients, insomnia began in childhood, while for 27.07% it began in adolescence. PCL-5 scores were associated with higher Insomnia Severity Index (ISI) scores, but not trauma. ISI scores were also higher for women, and positive relationships were observed between ISI scores, PCL-5 scores and the number of self-reported traumatic events among women.

**Conclusions:**

These exploratory results highlight that the relationship between PTSD symptoms and insomnia could be sex-specific. They also highlight the importance of PTSD symptoms screening for patients diagnosed with chronic insomnia.

## Highlights

- The prevalence of trauma and PTSD symptoms is high among (especially female) patients diagnosed with chronic insomnia.
- An evaluation of previous traumatic events and PTSD symptoms may help to improve insomnia care.
- PTSD screening may be important for patients diagnosed with chronic insomnia, as PTSD treatments could supplement insomnia treatments.

## Introduction

Insomnia is the most prevalent sleep disorder; it affects 15–20% of the adult population, especially women and the elderly (Zeng et al., 2020; Van Someren, 2021). Despite its prevalence and the acknowledged impact on socioeconomics and quality of life (Léger and Bayon, 2010), chronic insomnia is poorly recognized by public health authorities worldwide. This may be due to ongoing uncertainties about underlying mechanisms and drivers for developing the disease. In practice, its natural history remains relatively poorly understood, despite the existence of numerous models (Van Someren, 2021). Several hypotheses for its pathogenesis have been proposed in recent decades which highlight the wide range of determinants potentially involved in its development. It has been established that previous exposure to stress is a precipitant, and insomnia models based on stress diathesis provide a better understanding of the pathological processes involved, even if the pathophysiology of the disease remains insufficiently understood (Kalmbach et al., 2016, 2018).

The stress-based approach is consistent with one of the most popular models, namely the 3P model, which distinguishes Predisposing, Precipitating, and Perpetuating factors (Spielman et al., 1988; Wright et al., 2019). The latter biopsychosocial model suggests that *Predisposing* factors occur in the premorbid phase of the disease, and can be biological (genetic and epigenetic), social, or psychological (Wright et al., 2019). *Precipitating* factors are defined as clinical or psychologically significant events that interfere with the individual's sleep during a specific period of his or her life. The behaviors and thought patterns that are put in place in an attempt to compensate for or cope with difficult or stressful times are considered potential *Perpetuating* factors. The presence of the latter factors tends to support the development of a chronic condition, even when precipitating factors are no longer present.

Recent work has identified five clinical subtypes of insomnia disorder, as a function of the level of distress and sensitivity to reward on the one hand, and reactivity to the environment on the other (Blanken et al., 2019). The subtypes are based on a multivariate pattern of stable characteristics, such as life history, trait positive or negative affect, and personality, and are in line with the role of stress response dysregulation in sleep disturbance (Kalmbach et al., 2018; Hertenstein et al., 2019; Van Someren, 2021). Moreover, prospective epidemiological studies have hypothesized that high premorbid stress reactivity may be a trait-like diathesis of insomnia, and the process of sensitization may correspond to exposure to a major stressful life event (Nusslock and Miller, 2016).

In this context, the Adverse Childhood Events (ACEs) framework can be used to study such events. ACEs are potentially traumatic events that occur in childhood and adolescence (up to 18 years of age), and are known to be associated with stress sensitization (Nusslock and Miller, 2016). Their prevalence does not vary as a function of the patient's country or culture (Kessler and Ustun, 2008; Sacks and Murphey, 2018) and, regardless of age, women are more highly represented among those who have faced four or more ACEs. Conversely, for all ages, the percentage of men who have never faced an ACE is always higher than that of women (Rosenman and Rodgers, 2004; Dube et al., 2005).

Furthermore, there is a growing body of scientific knowledge that suggests an association between ACEs and multiple sleep disorders in adulthood (Kajeepeta et al., 2015; Lewin et al., 2019). The literature provides support for the idea that a high risk of lifetime stressors and ACEs can predict trauma and stress-related diseases such as post-traumatic stress disorder (PTSD) (Frewen et al., 2019). However, healthcare professionals have only recently taken the impact of ACEs on health problems in adulthood into account, despite the risk of PTSD (Kessler et al., 2010; Scott et al., 2010).

PTSD is a debilitating mental disorder that may develop after experiencing or witnessing a life-threatening event. It occurs in 5–10% of the population and is twice as frequent among women compared to men (Yehuda et al., 2015). Symptoms include re-experiencing, avoidance of situations that recall the event, negative alterations in cognition and mood, and increased arousal and reactivity (American Psychiatric Association, 2013). Although many patients complain of sleep disturbances (insomnia, nightmares, periodic leg movements, disruptive nocturnal behaviors, etc.) they are commonly assumed to be a secondary symptom of PTSD (Spoormaker and Montgomery, 2008; Hall Brown et al., 2015).

However, numerous clinical trials report a strong correlation between altered sleep and PTSD, and clinical reports show that sleep alterations often precede PTSD symptoms (Koffel et al., 2016). Research carried out in the past two decades highlights that insomnia is not only an epiphenomenon or consequence of other PTSD symptoms—rather, insomnia symptoms have been found to have an impact on the development and maintenance of other PTSD symptoms (Cox et al., 2017; Germain et al., 2017). From a psychological point of view, fear of sleep could maintain trauma-induced insomnia via fear-related arousal, especially in individuals with PTSD (Werner et al., 2021). From a physiological point of view, other authors argue that there is a reciprocal link between poor sleep, especially rapid eye movement (REM) sleep fragmentation, and PTSD (Saguin et al., 2021).

Against this background, our exploratory study examines the relationship between sex, chronic insomnia, self-reported traumatic life events, and PTSD symptoms.

## Materials and methods

### Participants

Our aim was to explore trauma history and PTSD symptoms in 50 new patients who complained subjectively of chronic insomnia according to ICSD-3 (American Academy of Sleep Medicine, 2014) criteria. Chronic insomnia was defined as a sleep disturbance that occurred at least three times a week and had been present for the preceding 3 months.

It was evaluated by a clinical interview carried out by sleep physicians during an initial consultation at our sleep and vigilance center at the Hôtel-Dieu hospital (Paris, France). The center is open to the public and is part of the French health service (Assistance Publique Hôpitaux de Paris). It brings together sleep physicians, psychologists, and psychiatrists who specialize in cognitive-based therapy and/or behavioral sleep medicine, and sleep technicians who specialize in the recording of sleep disorders and associated symptoms.

### Protocol and variables

Participation was proposed to patients by the sleep physician in charge of insomnia during the study period. For some, this occurred either following their initial consultation at the center or following several consultations if symptoms were still present despite treatment. In most cases, patients had been treated by other specialized sleep services but were still complaining of chronic insomnia. After being informed of the purpose of the study—to understand the relationship between trauma and insomnia—volunteer patients gave written informed consent. The Ethical Committee of Paris IDF 2 gave ethical approval.

Patients completed the following self-assessment questionnaires that took 20–30 min to fill in:

- **The Insomnia Severity Index (ISI)**. A seven-item self-report questionnaire that assesses the nature, severity, and impact of insomnia during the preceding month (Morin, 1993; Bastien et al., 2001). An ISI score ≥ 15 indicates clinically significant insomnia. Internal consistency was acceptable (Cronbach's alpha: 0.73).
- **The Trauma History Questionnaire (THQ**). A 24-item self-report measure that examines the individual's experience of potentially traumatic events, such as crime, disaster, and sexual and physical assault, using a yes/no format (Jehel and Ménager, 2000; Hooper et al., 2011). In some cases, traumatic experiences before and after the age of 18 overlap with ACEs. For each reported event, respondents were asked to record its frequency, and their age at the time it happened.
- **The PTSD Check List Scale** (PCL-5) (Blevins et al., 2015; Ashbaugh et al., 2016). The scale assesses the following four symptoms: re-experiencing (cluster B); avoiding situations that recall the event (cluster C); impairment of cognitive and emotional affect (cluster D); and increased arousal and reactivity (cluster E). Internal consistency was good (Cronbach's alpha: 0.91). Two methods were used to classify participants into no PTSD or provisional PTSD groups. First, we adopted the cut-off point proposed by the National Center for PTSD, namely a score ≥ 33. Second, we adopted the scoring method proposed by the United States Department of Veterans Affairs. The latter considers each item rated as “moderately” or higher, based on the following DSM-5 diagnostic rules for provisional PTSD: at least one item in clusters B and C are scored as “moderately” or higher, and two items in clusters D and E are scored “moderately” or higher (see https://www.ptsd.va.gov/professional/assessment/documents/using-PCL5.pdf).

### Statistical analysis

Analyses were performed using the Statistica^®^ (v7.1) software package. Data are described as mean ± standard deviation (SD) or as a percentage. Participants were categorized into two groups according to their PTSD symptoms status: those with a score equal to or above the PCL-5 cut-off of 33 (the provisional PTSD Group); and those with a score under the cut-off (the No-PTSD symptoms Group). Between-group comparisons were performed using Pearson's chi-square test for categorical variables, and Student's *t*-test for quantitative data, after checking that the distribution was normal. Correlations were run using Bravais-Pearson analyses.

The effect of PTSD symptoms status (PTSD symptoms or No-PTSD symptoms), self-reported trauma (yes or no), and sex on the ISI score was investigated using a dedicated Kruskal-Wallis non-parametric analysis if necessary, following the application of the Levene test of the equality of variances.

The relation between age at the first self-reported traumatic experience and the ISI and PCL-5 scores was assessed in the group that reported being exposed to a traumatic event. Between-group comparisons (self-reported trauma up to age 18, compared to after 18 years old) were performed using Pearson's chi-square test for categorical variables (PTSD status) and Student's *t*-test for quantitative data (ISI and PCL-5 scores).

The statistical threshold of significance was set at *p* < 0.05. A trend was considered when *p* < 0.10.

## Results

### Population

Of the 50 initially selected patients, full data were obtained for 43 participants: 29 women (67.44%) and 14 men (32.56%), with an average age of 50.98 (SD 14.63). Seven patients were excluded due to a secondary diagnosis of another sleep disorder (obstructive sleep apnea or restless leg syndrome, based on polysomnography). Of the final sample, 55.81% lived with a partner, and the remainder lived alone. All had at least a master's degree and 62.71% were working (Table 1).

**Table 1:** Main sample characteristics for the total population (*N* = 43), and for provisional PTSD (*n* = 23) and no-PTSD (*n* = 20) groups.

	**Total**	**Sub-groups**	
		**Provisional PTSD**	**No-PTSD**
	***N*** **(%)**	***n*** **(%)**	***n*** **(%)**
Sex (women/men)	24/19	15/8	12/8
**Marital status**			
Couple: married, French civil partnership, or other	24 (55.81%)	13 (56.52%)	11 (55%)
Single or divorced	19 (44.19%)	10 (43.47%)	9 (45%)
**Professional status**			
Working	28 (62.71%)	14 (60.8%)	13 (65%)
Retired or unemployed	15 (37.21)	9 (39.1)	7 (35)
	**Mean (SD)**	**Mean (SD)**	**Mean (SD)**
Age	49.2 (13.8)	48.66 (11.35)	49.73 (16.18)
ISI	15.58 (4.51)	18.21 (5.41)	18.95 (3.61)
PCL-5 total	33.45 (15.6)	48.49 (8.53)	18.42 (8.69)
Re-experiencing	9.17 (5.85)	14.06 (5.92)	4.28 (5.79)
Avoidance	3.21 (2.61)	4.52 (2.79)	1.9 (2.42)
Impairment of cognitive and emotional affect	10.39 (4.12)	16.19 (4.82)	4.59 (3.42)
Increased arousal and reactivity	10.68 (6.37)	13.72 (6.23)	7.64 (6.52)

### Insomnia

The mean ISI score was 18.58 (SD 4.51). For 11.6% of patients, their insomnia began in childhood, and for 27.07%, it began in adolescence. For the remainder, the mean onset was 9 years previously (SD 8.23). Regarding their treatment at the sleep and vigilance center, 48.8% were being treated with pharmaceuticals (antidepressants: 9.3%, anxiolytics: 18.6%, melatonin: 9.3%), and 38.23% used phytotherapy.

### Prevalence of trauma and PTSD symptoms

Only seven patients (16.3%) did not report any traumatic event, while 36 (83.7%) reported at least one event. Among the group exposed to trauma, 94.44% reported at least two events. Concerning sex, no difference was found between men and women regarding the presence of self-reported past traumatic events (*X*^2^ = 0.8; *p* = 0.37) or their number [H_(1, N = 43)_ = 1.67; *p* = 0.19]. Among those exposed to self-reported trauma, in 86.11% of cases, the event was related to crime, and in 60% of cases it was related to a general disaster. Eight women (27.6%) and four men (28.6%) reported at least one sexual assault. As for age at first trauma, half (50%) reported that it occurred in childhood or adolescence (up to 18 years of age), and the remainder reported that it occurred when they were older.

The mean PCL-5 score was 33.45 (SD 15.62). Based on a PTSD score threshold of >32, 53.49% of patients suffered from provisional PTSD. Small differences were observed when using the Department of Veterans Affairs classification. According to the latter classification, 51.2% of patients suffered from provisional PTSD. Two patients with a PCL-5 score > 32 did not meet the Department's criteria, and conversely, one patient with a score < 32 did meet the criteria. Based on a threshold of >32, no differences were found between men and women with respect to PTSD symptoms status (χ^2^ = 0.48; *p* = 0.83) or PCL-5 scores [H_(1, N = 43)_ = 0.002, *p* = 0.97]. Mean scores on the PTSD sub-scales were as follows: 9.17 (SD 5.85) for re-experiencing; 3.21 (SD 2.6) for avoiding; 10.39 (SD 6.74) for impairment of cognitive and emotional affect; and 10.68 (SD 6.38) for increased arousal and reactivity (Table 1).

### Associations between sex, trauma, and PCL-5 and ISI scores

Correlation analyses found no statistically significant association between ISI scores, PCL-5 scores, and the number of self-reported traumatic events for men (−0.41 < *r* < 0.4; 0.15 < *p* < 0.49). However, a moderate to strong correlation was identified for women (0.36 < *r* < 0.58; 0.001 < *p* < 0.058).

**Figure 1:**
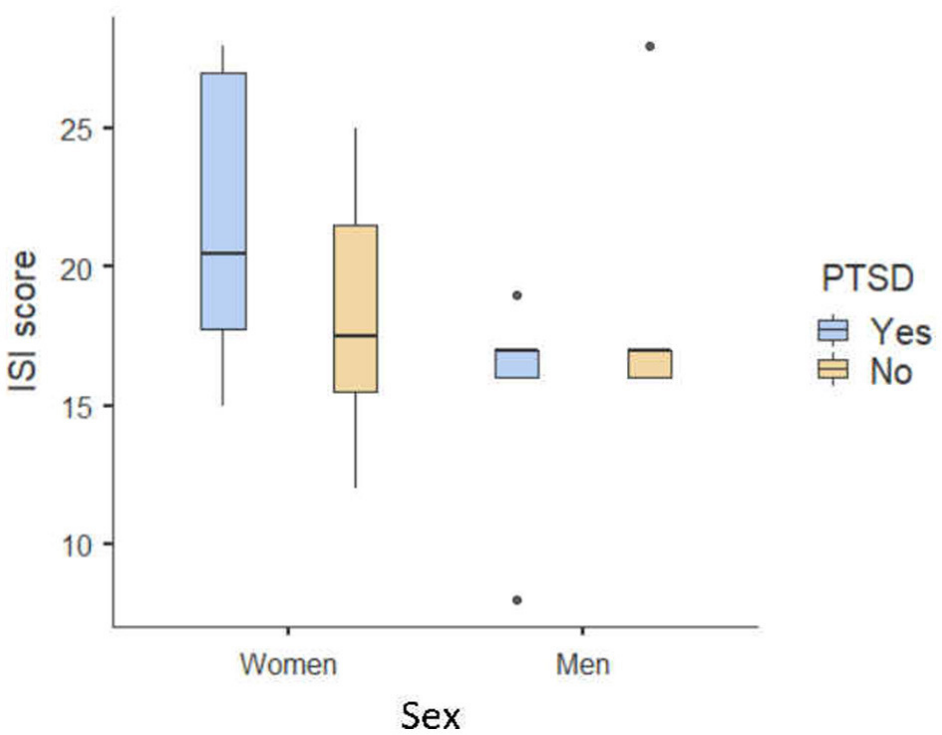
Box plot of mean ISI scores as a function of sex and PTSD status.

Kruskal–Wallis non-parametric analyses showed a significant effect of PTSD status [H_(1, N = 43)_ = 3.87 *p* = 0.05], a trend regarding past experience of trauma [H_(1, N = 43)_ = 3.32, *p* = 0.07], and an effect of sex [H_(1, N = 43)_ = 3.88, *p* = 0.048] on the ISI score. Patients with PTSD symptoms and female patients reported higher ISI scores than those without PTSD symptoms, and patients with experience of trauma tended to report higher ISI scores (Figure 1).

### Impact of the age of the first trauma on PTSD symptoms and ISI scores

No difference was found between the group that self-reported trauma up to the age of 18, and the group that reported trauma after the age of 18, with respect to PTSD symptoms status (χ^2^ = 0.48; *p* = 0.83). Among those who had experienced trauma, no difference was found with respect to the ISI score [H_(1, N = 36)_ = 1.47, *p* = 0.22] or the PCL-5 score [H_(1, N = 36)_ = 0.06, *p* = 0.81].

## Discussion

Our main finding is the high prevalence of historical traumatic events in our sample: 83.7% of patients diagnosed with chronic insomnia had experienced traumatic events, and over 50% suffered from provisional PTSD. Although other studies have reported similar results (Ashbaugh et al., 2016; Frewen et al., 2019), worldwide prevalence (24 countries; *n* = 68,894) of traumatic events has been assessed by the World Mental Health Survey (Kessler et al., 2017) as 70.4% over the course of a lifetime, with an average exposure of 3.2 traumas per inhabitant. Another study in the French population (Koenen et al., 2017), based on data from the World Mental Health Survey (26 surveys; *n* = 71,083), found a 72.2% rate of traumatic events, and a lifetime PTSD prevalence of 3.9%. The latter results are in accordance with the risk of PTSD associated with a history of ACEs (Frewen et al., 2019).

It has been suggested that traumatic insomnia could impede full recovery following exposure to extreme stress, leading to PTSD (Koffel et al., 2016). Furthermore, altered sleep quality pre- and post-trauma seems to be a mediating factor in PTSD (Saguin et al., 2021). In practice, altered sleep is considered not only as a predictive marker for PTSD onset, but also as a clinical marker for maladaptive stress in trauma responses, and even as a modifiable risk factor for PTSD relapse. The data suggest that a significant number of patients who suffer from PTSD, and who have been exposed to traumatic childhood events, initially present with complaints related to sleep disturbances (Koffel et al., 2016), and childhood trauma is considered to affect sleep health in adulthood (Brindle et al., 2018). The latter observation is in line with the growing body of evidence linking childhood trauma to adverse health outcomes later in life.

Among soldiers, both the number of types of traumatic stress and the number of traumatic events have been found to significantly predict the likelihood of developing PTSD (Brownlow et al., 2022). The latter finding highlights the important role of diverse, cumulative stress in the relationship between trauma and sleep disorders. However, to the best of our knowledge, there are no reports of a high rate of PTSD symptoms among patients suffering from chronic insomnia. In fact, the literature indicates that although exposure to trauma is common, only a minority of exposed individuals go on to develop PTSD (Hall Brown et al., 2015). Our results show that although PTSD scores were higher for patients who had been exposed to trauma, age at first trauma was not associated with a higher prevalence of PTSD or more severe symptoms.

Furthermore, it has been established that most patients with PTSD suffer from insomnia. In a recent meta-analysis of the prevalence of insomnia combined with post-traumatic stress symptoms (PTSS) (*n* = 573,665) the authors concluded that the prevalence of insomnia in PTSD/PTSS was 63% (CI: 45–78%), and was moderated by both the cause of the trauma and the score on the PTSD/PTSS scale (Ahmadi et al., 2022). The latter study also highlighted the importance of screening and managing insomnia in PTSD patients. Other authors have explored the physiological link between the severity of insomnia and the severity of PTSD (Saguin et al., 2021). Evidence supporting a reciprocal relationship between insomnia and PTSD highlights that several mechanisms could be involved (Ahmadi et al., 2022). The most relevant are shared genetic factors, maladaptive functioning of the endocrine system, hyperarousal, emotion dysregulation, and aberrant neural circuits, as these mechanisms are found in both PTSD and stress (Ahmadi et al., 2022). However, other authors suggest that the cause of the trauma may interfere with the history of PTSD (Hall Brown et al., 2015).

Whatever the mechanism, or the type and number of traumas, it is obviously desirable to be able to treat sleep disorders and insomnia in the management of PTSD (Coventry et al., 2020; Saguin et al., 2021; Ahmadi et al., 2022). It should be noted that if the analysis is expanded to life events that do not meet the criteria for traumatic events as described in the DSM-5 (American Psychiatric Association, 2013), the timing of the exposure, specifically whether it occurred during childhood, could be predictive of the risk of PTSD during the person's life (Frewen et al., 2019). These data provide support for the idea that a combination of traumatic and non-traumatic stressors across the lifespan, along with ACEs, have a particular impact on the development of symptoms associated with trauma and stress-related disorders (Frewen et al., 2019).

Our second finding is that none of our insomnia patients had been diagnosed with PTSD, and none were being treated for it. This suggests that it would be beneficial to educate practitioners about trauma during their Behavioral Sleep Medicine training. Furthermore, in the context of clinical care, our study highlights the importance of embedding psychologists and/or psychiatrists in sleep centers. Third, the use of cognitive behavioral therapy for insomnia could be completed by therapeutic approaches that address PTSD. The most scientifically robust treatments for PTSD are trauma-focused psychotherapies, including Prolonged Exposure, Cognitive Processing Therapy, Eye Movement Desensitization and Reprocessing, and antidepressants (NICE, 2018). With appropriate care, the remission rate is ~80% (NICE, 2018). Consequently, qualified sleep physicians could add these treatments to their therapeutic arsenal when treating patients suffering from chronic insomnia associated with PTSD.

Our third finding is that sex could influence the relationship between self-reported experience of trauma, PTSD symptoms, and ISI. Positive correlations between the number of self-reported traumas, PTSD symptoms, and ISI severity were only observed in women. Moreover, sex had an impact on ISI scores, with women reporting a higher subjective level of insomnia. However, the current results should be regarded as exploratory ones and it is important to recognize that the non-significance of correlation coefficients for men might be due to the smaller sample size of men than of women.

This exploratory study has several limits, notably the small sample size. Nevertheless, our results can be considered a proof of concept for further studies. The second limitation concerns the sociodemographic characteristics of the population, as men were under-represented. The latter important point may explain the observed differences in the significance of correlations between sexes. Given known differences with respect to PTSD, there is a need to evaluate in more detail the role of sex in the link between insomnia and PTSD symptoms severity. Third, we evaluated self-reported traumatic events with the THQ, as there is no validated French version of the ACE scale—however, the THQ is inadequate when evaluating the relationship between ACEs, PTSD, insomnia, and sex. There is a clear need to better investigate the role of ACEs in insomnia, as recent research has concluded that childhood adversity does not occur as a single entity but in clusters. Various studies have questioned the interpretation of the results of previous studies that consider a single childhood event as associated with a single mental illness.

Another limitation relates to the absence of comorbidity data, as it has been established that both insomnia and PTSD are frequently associated with comorbidities. Such associations may have had an impact on our results. Further studies need to take into account possible associations between insomnia, PTSD, and trauma history. Furthermore, it is known that poor sleep tends to be associated with certain sociodemographic and socioeconomic factors. Income and employment status are key socioeconomic determinants and are likely to explain the association between a low level of education and poor sleep (Grandner et al., 2010). Our population was highly educated, and hence unlikely to be representative of the population of patients suffering from insomnia. Finally, we used the ISI to evaluate insomnia. It would be interesting to include the duration of insomnia, along with other aspects of the person's sleep disorder, such as Trauma-Related Nightmares (TRNs) in further studies. Indeed, TRNs are the most common symptom of PTSD and are closely associated with its severity, treatment resistance, and chronicity (Nappi et al., 2012; Germain, 2013).

## Conclusion

Overall, our results highlight that women appear to be at higher risk of insomnia due to PTSD symptoms. It would be interesting to ask patients about how their insomnia began, whether it became worse at certain times in their life, and how it may be related to traumatic events. Our exploratory study highlights the importance of PTSD symptom screening among patients seeking treatment for insomnia, especially women, and this finding should be brought to the attention of sleep physicians. Even if we cannot yet directly claim that PTSD is the etiology of insomnia, it seems relevant to explore whether treating a patient for PTSD symptoms could help in the treatment of insomnia in cases where PTSD and insomnia are associated, and vice-versa.

## Data availability statement

The raw data supporting the conclusions of this article will be made available by the authors, without undue reservation.

## Ethics statement

The studies involving humans were approved by Ethical Committee of Paris IDF 2 France. The studies were conducted in accordance with the local legislation and institutional requirements. The participants provided their written informed consent to participate in this study.

## Author contributions

DL and MT designed the study. CG managed data quality control. FC, EL, and CR contributed to the data analysis. All authors actively took part in the process, planned and participated in the statistical analysis, and read and approved the final manuscript.
